# Perceived Differences in the Management of Mental Health Patients in Remote and Rural Australia and Strategies for Improvement: Findings from a National Qualitative Study of Emergency Clinicians

**DOI:** 10.1155/2011/965027

**Published:** 2011-05-02

**Authors:** G. A. Jelinek, T. J. Weiland, C. Mackinlay, N. Hill, M. F. Gerdtz

**Affiliations:** ^1^Department of Medicine, University of Melbourne, Fitzroy, Victoria 3068, Australia; ^2^Department of Emergency Medicine, St. Vincent's Hospital, Fitzroy, Victoria 3068, Australia; ^3^Department of Nursing, University of Melbourne, Fitzroy, Victoria 3068, Australia

## Abstract

*Introduction*. We aimed to describe perceptions of Australian emergency clinicians of differences in management of mental health patients in rural and remote Australia compared with metropolitan hospitals, and what could be improved. *Methods*. Descriptive exploratory study using semi-structured telephone interviews of doctors and nurses in Australian emergency departments (EDs), stratified to represent states and territories and rural or metropolitan location. Content analysis of responses developed themes and sub-themes. *Results*. Of 39 doctors and 32 nurses responding to email invitation, 20 doctors and 16 nurses were interviewed. Major themes were resources/environment, staff and patient issues. Clinicians noted lack of access in rural areas to psychiatric support services, especially alcohol and drug services, limited referral options, and a lack of knowledge, understanding and acceptance of mental health issues. The clinicians suggested resource, education and guideline improvements, wanting better access to mental health experts in rural areas, better support networks and visiting specialist coverage, and educational courses tailored to the needs of rural clinicians. *Conclusion*. Clinicians managing mental health patients in rural and remote Australian EDs lack resources, support services and referral capacity, and access to appropriate education and training. Improvements would better enable access to support and referral services, and educational opportunities.

## 1. Introduction

Despite rural and remote Australians having higher rates of mental illness, they lack specialist mental health services [1]. This is of concern to policy makers and practitioners, and to patients who report poor quality mental health services and prejudicial attitudes from staff in rural emergency departments (EDs). EDs are often the only places where acute mental health care is available 24 hours a day in rural centres. In Australia, these EDs are mostly staffed by general practitioners or career medical officers. Clinicians in these environments, both doctors and nurses, often have extensive generalist skills that their counterparts in urban practice do not have. However, there are often deficiencies in their confidence and skills in managing common mental health emergencies, and there have been calls for them to be better trained in this area [1]. 

There is limited availability of education and training for ED staff in the management of acute mental health conditions, particularly in rural areas. Despite recommendations for increased education to improve awareness of mental health among ED clinicians in general [2], structured education for ED clinicians is lacking, apart from specialised postgraduate courses, as is a clear identification of their learning needs. A learning needs analysis of Australian emergency physicians was conducted in 2006 via survey [3], but there were no data related to the issues around the mental health-related learning needs of doctors in rural and remote areas, and there has to date been no comparable study for nurses. 

As part of a national study into the learning needs of emergency doctors and nurses across Australia, this study aimed to describe these emergency clinicians' perceptions of whether there were differences in assessment and management of mental health patients in rural and remote areas in comparison to metropolitan hospitals and ways of improving the management of these patients in rural EDs.

## 2. Methods

### 2.1. Study Design and Ethics Approval

A descriptive exploratory study was undertaken, based on semistructured telephone interviews. This study was approved by the Human Research and Ethics Committees at La Trobe University's Faculty of Health Sciences.

### 2.2. Interview Schedule Design and Development

A national learning needs analysis of ED clinicians was undertaken, overseen by a research team at St Vincent's Hospital Melbourne with responsibility for questionnaire design, data collection, and data analysis. The interview schedule was developed through an iterative process by this team of two emergency physicians, a researcher/emergency nurse, a research psychologist, and a research officer.

The final interview schedule (Table 1) included one closed question eliciting yes/no/undecided responses to a series of potential learning areas and 16 open-ended questions on various aspects of the learning needs of ED clinicians in managing mental health patients in the ED, including confidence, attitudes, differences between city and rural settings, the effect of resources and comorbidities on assessment, mental health triage, and areas to prioritise for learning. 

The current study reports those data from ED clinicians related to direct questioning (yes/no/unsure) eliciting responses to the question of whether the clinicians perceived differences in the assessment and management of mental health patients in rural and remote areas in comparison to metropolitan hospitals, and what they were. Australia's population is concentrated in the major capital cities, and most other towns are relatively small. Clinicians in remote and rural areas in this study were mostly working in smaller rural hospitals. The clinicians were also asked for ideas for improving the management of these patients in rural EDs that may not have access to ED-based clinicians specialising in mental health. While some of the other open-ended questions also elicited qualitative responses related to subthemes within the broad themes discussed under these questions, they were not specifically related to differences between rural and urban settings and so were not further analysed. 

We felt that both clinicians working in remote and rural work contexts and those in city hospital environments could provide useful and potentially contrasting views in response to these research questions and so incorporated data from both groups in this study.

### 2.3. Participants

In August 2009, doctors who were members of the Australasian College for Emergency Medicine (ACEM) and nurses who were members of the College for Emergency Nursing Australasia (CENA) were invited to be interviewed. Staff in these groups were considered for inclusion if they were working clinically in Australian EDs at the time of the study. Participants were selected from those responding in accordance with attempted proportional distribution across regional and metropolitan settings and across Australian states and territories where possible.

### 2.4. Recruitment and Data Collection

ACEM and CENA distributed an email invitation to their members on behalf of the investigators to participate in this study. Interested members were invited to email the research officer to express their interest. The research officer then scheduled a time for telephone interview. The Plain Language Statement and interview questions were emailed to the participant prior to the scheduled interview, and all participants provided written informed consent. Interview responses were audio-recorded to permit later verbatim transcription. Transcriptions were provided to participants for validation. Interviews were concluded in February 2010.

### 2.5. Data Analysis

After transcription and validation, content analysis of qualitative data was undertaken by two research officers using the Framework Method by Ritchie and Spencer [4]. Themes and subthemes were determined independently by two members of the research team, and consensus reached before apportioning responses. Themes and subthemes were analysed according to clinical role (doctor or nurse).

## 3. Results

### 3.1. Participant Characteristics

In total, 39 ACEM members and 32 CENA members responded to the invitation, from which a sample of 20 ED doctors (51% of those responding) and 16 ED nurses (50% of those responding) was selected for participation based on clinical role and jurisdiction. The sample was stratified according to metropolitan and rural EDs, Australian states and territories and seniority of position held in the ED.  Table 2 demonstrates the participant's' characteristics. 

### 3.2. Differences in Management of Mental Health Patients in Rural and Remote EDs

In response to direct questioning about whether the clinicians perceived differences in the assessment and management of mental health patients in rural and remote areas in comparison to metropolitan hospitals, and what they were, responses from the 36 clinicians fell into 3 broad themes, resources/environment, staff, and patient issues. 

Under resources/environment, 14 doctors and 10 nurses offered responses. Both groups of clinicians noted limited access in rural areas to psychiatric support services (8 doctors, 9 nurses); alcohol and other drug services (7 doctors, 2 nurses); limited options for referral (3 doctors, 2 nurses). Doctors only reported that the logistics of referral presented challenges (1 doctor) and nurses only commented specifically on limited resources generally (6 nurses). 

Under the theme of staff issues, comments came from 11 doctors and 7 nurses, under the following subthemes. Both groups of clinicians noted a lack of knowledge and understanding of mental health issues in rural areas (6 doctors, 5 nurses); differences in the assessment and management of rural mental health patients (8 doctors, 1 nurse); a lack of acceptance of mental health conditions in rural areas (3 doctors, 1 nurse); a lack of understanding about dual (physical and mental health) diagnosis (1 doctor, 1 nurse). Doctors noted that staff are more resilient (5 doctors) and flexible (1 doctor) in rural EDs.

Under patient issues, 3 doctors and 2 nurses made comments. They noted a better understanding of social issues contributing to mental health problems for rural patients from local communities (3 doctors, 1 nurse); similarly better community and family support (1 doctor, 1 nurse), although 1 doctor felt there was actually less of this support. 

Three doctors and 1 nurse provided responses indicating they were not sure if there were differences or that the question was not applicable as they had not worked in the country. 

Qualitative responses below are listed with remote and rural practitioner responses first, followed by relevant responses from city practitioners.

### 3.3. Suggested Improvements in the Management of Mental Health Patients in Rural EDs

In response to direct questioning asking for ideas for improving the management of these patients in rural EDs that may not have access to ED-based clinicians specializing in mental health, 1 doctor and 3 nurses stated they had no rural experience and did not offer other responses. Otherwise, responses from the 36 practitioners fell into three broad themes of resources, education, and guidelines. 

Under the theme of resources, 17 doctors and 10 nurses offered specific responses, with the following subthemes. Medical and nursing clinicians suggested improvements in access to mental health experts for consultation (8 doctors, 4 nurses); improving access to specific mental health services in rural areas (4 doctors, 5 nurses); establishing support service networks (4 doctors, 1 nurse); implementing specific mental health services in rural areas (2 doctors, 3 nurses). Only doctors offered suggestions about ensuring access to mental health teams/experts (7 doctors); increasing resources to assist in the management of aggressive patients (1 doctor); enhancing visiting psychiatric provider coverage (1 doctor); enhancing existing mental health services in rural areas. Only nurses offered suggestions about improving links between ED staff and mental health staff (2 nurses); implementing case management approaches (1 nurse). 

Under the theme of education, 10 doctors and 5 nurses made a range of specific suggestions, grouped into these subthemes. Both groups of clinicians made suggestions about training local clinicians to improve MH knowledge and access to local expertise (6 doctors, 2 nurses); education about patient assessment and management (6 doctors, 1 nurse). Doctors only had ideas about risk assessment training (3 doctors); acute sedation and psychiatric medication training for GPs working in ED settings (2 doctors); training to manage acute agitation (1 doctor); education about sedation (1 doctor); training in communication techniques (1 doctor); the provision of workshops specific for rural and remote clinicians working in EDs; specific education about mental health legislation (1 doctor). Nurses only made suggestions about appropriate referral pathways (1 nurse); education about common ongoing mental health issues (1 nurse). 

Under the theme of guidelines, 3 doctors and 4 nurses offered specific suggestions, with the following subthemes. Medical and nursing clinicians suggested utilising best practice guidelines (1 doctors, 3 nurses); developing supporting information and best practice resources (2 doctors, 1 nurse).

Qualitative responses below are listed with remote and rural practitioner responses first, followed by relevant responses from city practitioners.

### 3.4. Doctors' Responses

In terms of differences between rural and metropolitan management, rural and metropolitan doctors agreed that a common theme was lack of access to support services in rural EDs; doctors acknowledged the difficulty of practising in this environment:

*“I think that [mental health management in a rural environment] is really very very hard …” (rural ED registrar)*

*“The access to psychiatric services … can be very variable in parts and accessing an opinion can be difficult and that leads to people either over-calling a situation … sticking sedated patients on a plane and flying them several thousand kilometres to see a psychiatrist in Perth, or under-calling it and taking a risk that the patient is going to be safe in the community.” (rural deputy ED director)*

*“I've certainly worked in places where there were little or no psychiatric services and the main problems are having to arrange and get someone to accept a patient …. In cases where there's no retrieval service you're looking at tag-teaming ambulances, need to organise police and there can be a major drama.” (rural emergency physician)*

*“In a tertiary centre where you can just say “he needs to be detained and sedated or sorted out in the morning” as opposed to when you really haven't got that option, or it's much more difficult and complex …” {metropolitan ED registrar}*

*“I think the assessment is very similar, I think the management would be very different. Because for acute people that need admission, it's very difficult to admit appropriately in rural areas if you don't have facilities. And when you try to contact the big hospital, they go “oh we're full”. ” (etropolitan emergency physician)*

*“… there's often long distances in between where there might be a bed actually available for a patient if they need to come in as an inpatient, and so therefore that takes the patient out of their immediate support zone with people able to visit …”. (metropolitan ED director)*

*“… trying to get tertiary advice sometimes … the problem is often you're talking to a junior doctor …. So it's actually around having good networks and supports and a clear chain of command around who you need to talk to.” (metropolitan ED director)*

*“One of the things that is difficult in those situations is security, because you don't have security guards to come in and help you.” (metropolitan ED registrar)*

Although others were not so sure that more services meant better care:

*“… it's not always the case that bigger is better. There are some quite dysfunctional relationships in big cities …” (rural ED director)*

There were comments about better community support and local knowledge in rural areas:

*“The other thing that's good about rural … you do have more access to family and community supports.” (metropolitan ED registrar)*

*“They might have the benefit of actually knowing their patients better, and knowing the community better; knowing how the family are coping, or whether the family will cope with the patient or whether they do need an admission or discharge.” (metropolitan emergency physician)*

Staff issues were mentioned:

*“… the guys who work in the rural [areas] tend to be a little bit more resilient, a little bit more flexible and more prepared to do things which are outside of so-called call, as they simply have to.” (rural emergency physician)*

In terms of patient factors, attitudinal issues in rural areas were raised by some doctors:

*“… for more rural settings mental health is less well understood, is more of a taboo, and I think more difficult for patients …” (rural ED registrar)*

*“… some of the prejudices that are barriers to effectively managing them, I think they're a bigger problem in the rural centre than in central centres …” (rural ED director)*

Suggestions for improving the management of mental health patients in rural areas again focused on resources:

*“… would be good … for people to actually have access to a central number.” (rural emergency physician)*

*“… physical and online resources … would be helpful.” (rural emergency physician)*

*“… psych liaison services available more frequently in those places …” (rural ED director)*

*“… one of the things they've brought in for that is actually tele-medicine …” (rural ED registrar)*

*“… it would be useful if they could have at least a hotline they could speak with a experienced clinician …” (metropolitan ED registrar)*

*“Having a 1800 number where you know you're going to speak with a psychiatrist rather than a junior registrar …” (metropolitan ED director)*

*“… it's trying to get better links and better support services from the regional hospitals in the area; but one, it's a matter of staffing; two, it's a matter of distance. I don't think I've got any easy solutions for them.” (metropolitan emergency physician)*

*“… it does help if they can have access to the mental health database …” (metropolitan emergency physician)*

A common theme was increasing skills, particularly for acutely agitated patients:

*“… one of the crucial and key points is increasing the skills in the emergency management of the acutely agitated psychiatric patient …” (rural deputy ED director)*

*“… increasing the assessment and management skills of both experienced nurses or nurse practitioners and the ED medical officers.” (rural deputy ED director)*

*“… doctors and nurses [working rurally] quite frequently go to two-day workshops like the ELS or the EMST or the ALS. What's really quite interesting is there is no equivalent course for acute mental health illness, or acute mental health presentations to ED.” (rural emergency physician)*

*“Having them skilled up, particularly in acute psychiatric presentations, would be probably one of the best things.” (metropolitan emergency physician)*

*“If you don't have abilities to actually manage somebody's airway, then you are much more restricted in what you can actually give a patient to try and calm them down if you feel you need to …” (metropolitan ED director)*

*“… need increased education to assess and diagnose and properly manage mental health diagnoses and patients who do have mental health problems. … one way to do that could be to have some online resources …. The other thing … is whether there are any guidelines that they can access on line.” (metropolitan ED registrar)*

### 3.5. Nurses' Responses

Again, resource issues dominated the discussion of differences between the country and city, both by rural and city clinicians:

*“… it all comes down to resources.” (rural)*

*“… obviously the metro are far better staffed to have greater resources than the rural do …” (rural)*

*“And with the drug and alcohol—it's just one person organising and coordinating it all …” (rural)*

*“… it's a little bit different being in a small rural hospital than metropolitans because people don't tend to hospital shop. They just tend to come to this one hospital.” (rural)*

*“… historically it's difficult to recruit into those positions.” (rural)*

*“… we have limited health clinicians.” (rural)*

*“… there's not many places that you can get help from in a rural setting.” (rural)*

*“We've got back up from the police in the event of an aggressive client too so although we don't have the skill to do counselling we have back up for safety.” (rural)*

*“… they don't have the same resources … and that tends to be a major stumbling block for them because they often have to transfer patients out that maybe didn't need to be transferred out because they don't have someone local or even close by …to … service the patients' needs.” (metropolitan)*

*“Lack of resources; lack of clinicians for sure; lack of education for ED staff to manage these people.” (metropolitan)*

*“… there needs to be better services out there for those sort of people …” (metropolitan)*

*“I've worked there … it's the lack of resources, a lack of education …” (unspecified)*

*“… they're very, very heavily reliant on the police to assist them with anything …” (metropolitan)*

Suggestions for improving rural services included better resourcing and access:

*“… definitely psychiatric care was something that they needed to plan to come through not necessarily on a daily basis, but at least coming through town regularly, weekly/two-weekly.” (rural)*

*“I think having telephone psych support for staff in rural EDs would be really useful …. Not necessarily to psychiatrists, but to someone like psych triage or a psychiatric nurse, community health services that are able to hook patients up with, community mental health services.” (metropolitan)*

*“… as much as possible, standardised ways of dealing with people and also 24-hour consultation access.” (metropolitan)*

Some suggested better case management:

*“… more case management and more management plans in place …. we have our regulars that come in, and if management plans are in place, it does make it a bit easier.” (rural)*

And better training was again a theme:

*“… we had no real training for mental health or no real understanding how at least look after them …. they are now teaching part of mental health in the ETEK training program, the triage training program nationally approved.” (rural)*

*“Train and broaden the scope of RNs out there, similar to like a nurse practitioner who can suture and stuff, maybe they can broaden the scope of an NP …, broaden the RN role with some specific [mental health] training …” (unspecified)*

*“… making sure that post graduate and under graduate education covers mental health.” (metropolitan)*

## 4. Discussion

Respondents in this study nominated constraints in resources, lack of psychiatric support services, particularly alcohol and other drug services, and limited options for referral as major differences in the rural and remote environment. Further, there was an acknowledgment of deficiencies in skills and knowledge, to some extent balanced by the resilience and flexibility of clinicians in these settings. A number of respondents reported concerns about attitudes towards patients with mental health problems amongst clinicians in rural EDs.

Despite some advantages in having local knowledge of the social and family circumstances of patients presenting with mental health problems in rural and remote areas, our study showed that ED clinicians face very significant barriers to the delivery of effective mental health care because of lack of resources, lack of training, and lack of easy access to specialist support. Many related anecdotes of particularly difficult cases, mostly centred around acutely agitated patients requiring chemical restraint and transfer and the associated logistic difficulties. Responses from both metropolitan clinician groups indicated significant concern and sympathy for their rural colleagues' predicament, delivering mental health care in an underresourced isolated environment, often with inadequate training.

In suggesting ways to improve the situation, a range of responses was provided; most were realistic in suggesting innovative ways of accessing specialist support, rather than hoping for increased government expenditure for local resources and infrastructure, and optimizing training and skills for local rural clinicians. A number of participants suggested a dedicated mental health line with easy access to senior specialist psychiatric opinion, and this would appear to be an option worthy of further investigation and research. Others noted the potential of internet-based conferencing and training packages, and material on clinical guidelines, referral pathways, and appropriate forms for patient detention and transfer. 

There were suggestions that a weekend course for emergency management of mental health presentations could be developed and delivered in rural locations, similar to the Emergency Life Support Course offered by the Australasian Society for Emergency Medicine, or the Early Management of Severe Trauma Course offered by the Royal Australasian College of Surgeons. A number of organizations, such as the Discipline of Emergency Medicine at the University of Western Australia, in conjunction with Rural Health West, now conduct rural weekend courses in cardiac and respiratory care, orthopaedic emergencies, toxicological emergencies, and others [5]. These locally delivered courses have been extremely well received by practitioners in rural and remote locations, and a mental health emergencies course could be modeled along similar lines.

A mental health care course specifically designed for rural and remote practitioners has been evaluated in Western Australia [1]. The Managing Mental Health Emergencies course improved confidence, skills, and attitudes, enabling participants to better differentiate between substance intoxication and psychosis, and dementia and delirium, themes identified in our research. Thematic analysis in follow-up interviews revealed that participants recognised their prejudicial attitudes, developed new skills they were putting into practice, and communicated better. Our study clearly identifies a need for such courses to be rolled out to rural and remote practitioners; these may address some of the concerns we identified about the need for attitudinal change in rural EDs towards patients seeking mental health care. 

Online programs appear to offer some advantages. The Mental Health Emergency Care (MHEC) online learning program was recently trialed in several rural New South Wales health service locations [6]. Modeling collaborative practice in emergency mental health care, this program was shown to improve confidence and perceived effectiveness in managing acute mental health problems. There is clearly great scope here for innovative and effective delivery of emergency mental health care education to rural and remote areas, as suggested by many participants in our study. 

There is limited research on the benefits of bolstering acute mental health services in rural Australia. A regional/rural crisis assessment and treatment service (CAT) was trialed in rural Western Australia in a before and after study measuring its impact on inpatient admissions to an acute adult psychiatric facility [7]. While there was a non-significant trend in this study towards fewer and more appropriate admissions, policy makers and state governments have historically been unwilling to commit significant resources to these areas despite the clear need. Enhancing training opportunities is likely to be seen as more practical and cost-effective. 

### 4.1. Limitations

Despite around a third of the respondents in this study who provided demographic data are working in rural and remote areas, our data may have been somewhat biased towards metropolitan attitudes to mental health care in rural areas. Many of the metropolitan respondents, however, indicated that they had worked in rural environments previously. The sample was broadly representative across jurisdictions, region, and grade of medical staff, but unfortunately there were no nurses from New South Wales or clinicians from the Australian Capital Territory in the study. This limits the generalisability of the results.

## 5. Conclusion

Australian clinicians working in rural and remote EDs face significant hurdles in delivering acute mental health care in comparison with their city counterparts, particularly in lack of resources, support services and referral capacity, but also in access to appropriate education and training opportunities. There is a compelling case for improving ease of access to support and referral services, and providing appropriate educational opportunities, either with locally delivered and tailored courses or via online learning.

## Figures and Tables

**Table 1:** Identifying the mental health learning needs of emergency clinicians: interview schedule.

Domain	Question and prompt
Scope of practice	(1) Can you tell me what your role in managing MH patients in the ED and what services or resources (if any) are in place in the ED to assist you?
	(2a) {ASK ONLY IF RESOURCES ARE PRESENT} How do these ED based resources or services impact on your understanding of MH?
	(2b) {ASK ONLY IF RESOURCES ARE PRESENT} And how do these resources affect the assessment or management of mental health patients?
	(3) What do you think are the barriers to the effective assessment & management of MH patients in the ED?
	(4) What ideas do you have for improving the assessment & management of MH patients in your ED?
	(5) What ideas do you have for improving the management of MH patients in rural EDs that may not have access to ED-based clinicians that specialise in mental health?

Competence	(6a) What knowledge deficits (if any) do you feel that you or other ED clinicians have?
	(6a) Do you think ED clinicians would be interested in learning more about these areas (why/why not)?
	(7) What factors do you think impact on the accuracy of triage for mental health patients?
	(8) What are the barriers to enforcing mental health legislation in the ED?
	(9) Can you tell me about your experiences of mental health training specific to the ED?

Confidence in managing MH patients in the ED	(10) What factors affect your confidence in assessing MH patients?
	(11) In which areas do you feel least confident in treating MH patients?

**Table 2:** Participant characteristics.

Characteristic	ED doctors	ED nurses
	(*n* = 20)	(*n* = 16*)
Jurisdiction: *n* (%)		
Victoria	5 (25%)	4 (29%)
New South Wales	6 (30%)	0 (0%)
Western Australia	3 (15%)	4 (29%)
Queensland	3 (15%)	3 (21%)
South Australia	1 (5%)	3 (21%)
Northern Territory	1 (5%)	0 (0%)
Tasmania	1 (5%)	0 (0%)
Australian Capital Territory	0 (0%)	0 (0%)

Region: *n* (%)		
City/metropolitan	13 (65%)	11 (79%)
Rural/regional	7 (35%)	3 (21%)

Position held: *n* (%)		
ED Director	4 (20%)	
Deputy ED Director	1 (5%)	
Emergency Physician	8 (40%)	
Emergency Registrar	7 (35%)	

*2 nurses provided no demographic data.
